# Spatial and Temporal Pattern of Rift Valley Fever Outbreaks in Tanzania; 1930 to 2007

**DOI:** 10.1371/journal.pone.0088897

**Published:** 2014-02-25

**Authors:** Calvin Sindato, Esron D. Karimuribo, Dirk U. Pfeiffer, Leonard E. G. Mboera, Fredrick Kivaria, George Dautu, Bett Bernard, Janusz T. Paweska

**Affiliations:** 1 National Institute for Medical Research, Tabora, Tanzania; 2 Department of Veterinary Medicine and Public Health, Sokoine University of Agriculture, Morogoro, Tanzania; 3 Southern Africa Centre for Infectious Disease Surveillance, Morogoro, Tanzania; 4 Royal Veterinary College, London, United Kingdom; 5 National Institute for Medical Research, Dar es Salaam, Tanzania; 6 Food and Agriculture Organization of the United Nations, Dar es Salaam, Tanzania; 7 Department of Disease Control, University of Zambia, Lusaka, Zambia; 8 International Livestock Research Institute, Nairobi, Kenya; 9 Center for Emerging and Zoonotic Diseases, National Institute for Communicable Diseases, of the National Health Laboratory Service, Sandringham, South Africa; 10 School of Pathology, Faculty of Health Sciences, University of the Witwatersrand, Johannesburg, South Africa; The University of Texas Medical Branch, United States of America

## Abstract

**Background:**

Rift Valley fever (RVF)-like disease was first reported in Tanzania more than eight decades ago and the last large outbreak of the disease occurred in 2006–07. This study investigates the spatial and temporal pattern of RVF outbreaks in Tanzania over the past 80 years in order to guide prevention and control strategies.

**Materials and Methods:**

A retrospective study was carried out based on disease reporting data from Tanzania at district or village level. The data were sourced from the Ministries responsible for livestock and human health, Tanzania Meteorological Agency and research institutions involved in RVF surveillance and diagnosis. The spatial distribution of outbreaks was mapped using ArcGIS 10. The space-time permutation model was applied to identify clusters of cases, and a multivariable logistic regression model was used to identify risk factors associated with the occurrence of outbreaks in the district.

**Principal Findings:**

RVF outbreaks were reported between December and June in 1930, 1947, 1957, 1960, 1963, 1968, 1977–79, 1989, 1997–98 and 2006–07 in 39.2% of the districts in Tanzania. There was statistically significant spatio-temporal clustering of outbreaks. RVF occurrence was associated with the eastern Rift Valley ecosystem (OR = 6.14, CI: 1.96, 19.28), total amount of rainfall of >405.4 mm (OR = 12.36, CI: 3.06, 49.88), soil texture (clay [OR = 8.76, CI: 2.52, 30.50], and loam [OR = 8.79, CI: 2.04, 37.82]).

**Conclusion/Significance:**

RVF outbreaks were found to be distributed heterogeneously and transmission dynamics appeared to vary between areas. The sequence of outbreak waves, continuously cover more parts of the country. Whenever infection has been introduced into an area, it is likely to be involved in future outbreaks. The cases were more likely to be reported from the eastern Rift Valley than from the western Rift Valley ecosystem and from areas with clay and loam rather than sandy soil texture.

## Introduction

Rift Valley fever (RVF) is an arthropod-borne viral zoonotic disease caused by RVF virus (RVFV) belonging to the genus *Phlebovirus* of family *Bunyaviridae* [1], [2]. The disease is endemic in sub-Saharan Africa [3]–[5], but it has been reported outside this region [6]–[8] and it is considered to have potential for global spread [9]–[11]. The disease affects primarily domestic ruminants and humans [12]. The capacity of RVFV to cause large and severe outbreaks in animal and human populations and to cross significant natural geographic barriers, as exemplified by the virus spread over the Indian Ocean, Sahara desert, and the Red Sea in the past 3 decades, is of great concern for veterinary and public health authorities worldwide. RVFV is one of the most important emerging zoonotic threats, particularly to vulnerable African communities with low resilience to economic and environmental challenges [11]–[15].

There are significant differences in the ecology and transmission patterns of RVFV in endemic regions. In eastern and southern Africa large outbreaks of RVF occur at irregular intervals of up to 15 years, after heavy rainfall and floods [13]–[15]. After flooding of aedine mosquitoes breeding habitats (dambos), they are succeeded by *Culex* spp., which if infected upon feeding on viraemic vertebrate hosts further disperse the virus.

Recent molecular epidemiology studies indicate ongoing RVFV activity and evolution during the inter-epidemic period (IEP) in endemic areas and highlight the importance of a cryptic enzootic transmission cycle that allows for the establishment of RVFV endemicity and to precipitate explosive outbreaks [16], [17]. The RVFV transmission during IEP without noticeable outbreak or clinical cases has been reported in different species of African wildlife [18]–[20], in cattle in Mayotte [21], in sheep and goats in Mozambique [22], in humans in Tanzania [23], Kenya [15], and Gabon [24]. It has been postulated that during IEP, the virus persists in eggs of floodwater *Aedes* mosquito species or via low-level transmission between mosquitoes and vertebrates [12].

Host susceptibility depends on age and animal species. Young lambs, calves and kids are highly susceptible to infection with RVFV. In young lambs, the common signs include sudden rise of body temperature to 40.5–42.2°C, followed by death within 36 hours. Clinical signs in adult sheep and goats are not consistent but may include rise in body temperature, vomiting, mucopurulent nasal discharge, unsteady gait and high abortion rate up to 100% amongst pregnant ewes as well as haemorrhages manifestations. Clinical signs in adult cattle include high temperature, salivation, anorexia, general weakness, fetid diarrhoea, a rapid decrease in lactation and abortion. Abortion may be the only marked sign in cattle and mortality in adult cattle is usually less than 10% (25). The majority of infections in humans are unapparent or associated with moderate to severe, non-fatal, flu-like febrile illness with headache, nausea, myalgia and arthralgia [12]. Less than one percent of human patients develop the haemorrhagic and/or encephalitic forms of the disease. The overall case fatality ratio is estimated to range from 0.5% to 2%, but it appears to be higher in recent outbreaks of the disease in East Africa and South Africa [26]–[28]. In a minority of patients the disease is complicated by the development of ocular lesions [12].

Diagnosis of RVF is commonly carried out using enzyme linked immunosorbent assay (ELISA) methods that detects type-specific anti-RVFV immunoglobulins [29]–[33] and polymerase chain reaction (PCR) that detects RVFV nucleic acid [12] in blood. There is no specific treatment for RVFV infection in humans and animals and therefore management of clinical cases is only through supportive therapy [34].

Unpublished records available at the Ministry of Livestock and Fisheries Development (MoLFD) in Tanzania indicate that RVF-like disease in domestic ruminants occurred for the first time in 1930. RVF was added to the list of notifiable diseases under the Tanzanian Animal Disease Act in 1980 [35] and to the Integrated Disease Surveillance and Response (IDSR) Guidelines of the Tanzania Ministry of Health and Social Welfare in June 2011 [36]. RVF outbreaks have severe socio-economic impacts in Tanzania [37], [38]. For instance the last disease outbreak in 2006–07 had major adverse effects on rural household livelihoods, food security and nutrition [37]. In addition to pastoralists, the disease threatened the livelihoods of those who depend on livestock products and related activities for labour opportunities [37]. Past disease outbreaks in the country led to cessation of trade in ruminants. This resulted in serious economic losses for the sections of the population which were dependent on this source of income. Animals dropped in monetary value by 34%; the monthly internal market flow of livestock reduced by 37% and the annual external market flow by 54%. Loss due to death of domestic ruminants was estimated to be more than US$ 6 million. The Government spent about US$ 4 million for the control of the disease in 2006/2007 [37].

Despite the long presence of RVF in Tanzania, its spatial and temporal pattern has not been analysed. The objective of this study is to describe RVF outbreaks that have occurred in Tanzania between 1930 and 2007 and investigate the potential presence of clustering of cases during past outbreaks. We also aimed at investigating the association of the various environmental risk factors with the occurrence of reported outbreaks at district level during the last outbreak wave in 2006/2007.

## Materials and Methods

### Ethics Statement

This study was approved by the Tanzania Medical Research Coordinating Committee of the National Institute for Medical Research (NIMR/HQ/R.8a/Vol.IX/1296).

### Description of the Study Area

The United Republic of Tanzania, made up of Tanzania Mainland and Zanzibar, is located between longitude 29° and 41° East and latitude 1° and 12° South. The country experiences two major rainy seasons, namely a long rainy season during the months of March through to May and a short rainy season during the months of October through to January. The total annual rainfall ranges from 750–1400 mm. The dry season is mainly from July to September. A large central plateau makes up most of the mainland, at between 900 m and 1800 m above sea level.

**Figure 1:**
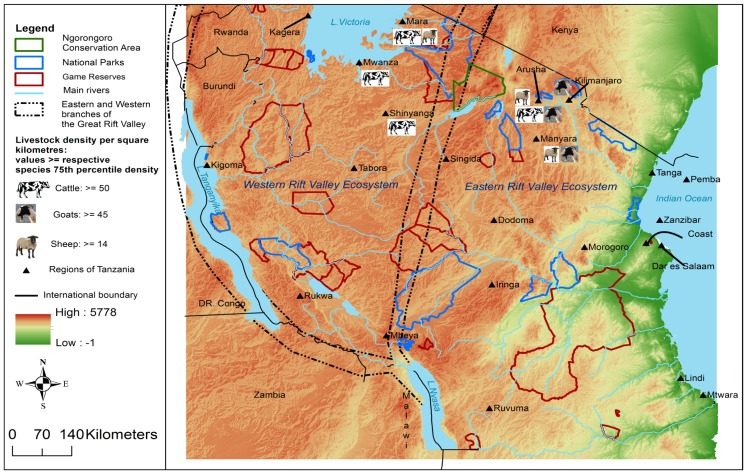
Digital elevation map of Tanzania and main features. Map of Tanzania showing the elevation (metres above sea level), Eastern and Western Great Rift Valley ecosystems (dashed-dot lines), main regions, main rivers, National Parks, Game Reserves, Ngorongoro Conservation Area, international borders and neighbouring countries. Rift Valley fever occurrence is associated with the Great Rift Valley. The animal symbols on the map indicate the areas where the density is equal to and above the threshold mentioned in the legend. Livestock density is higher in the plateau of the northern and lake zones of the country.

Pastoralism is mainly concentrated in the northern zone (Arusha and Manyara regions) and agro-pastoralism in the western zone (Tabora region), Lake Victoria zone (Shinyanga and Mwanza regions) and central zones (Dodoma and Singida regions) [39], [40]. The plateaus of the northern and lake zones are comprised of relatively higher livestock density; cattle ≥50, goats ≥45 and sheep≥14 heads per square kilometre (Figure 1). Spatial data for the past outbreaks was available at district and village resolutions. For administrative purposes each region in Tanzania is subdivided into districts which are further sub-divided into divisions, wards and villages. Districts are the smallest administrative units responsible for human and livestock disease management. To allow an analysis across the whole period, outbreaks were geo-referenced according to the administrative arrangement consisting of 21 regions, 120 administrative districts and 9504 villages during the latest disease outbreak in 2006/2007 [41].

RVF is associated with the Great Rift Valley system which is a long depression in the earth that runs down the eastern side of Africa. It extends from Syria in the Middle East, right down to Mozambique in south-eastern Africa. The well-expressed Eastern branch (flunk) traverses Ethiopia (Main Ethiopian Rift), Kenya (Kenya or Gregory Rift) and reaches north Tanzania, where it forms the north Tanzania divergence (Figure 1). Tanzania is the only country with two branches of this system forming the eastern and western ecosystems with one flunk branching at the south-western tip of Tanzania and running through the western, periphery of both Tanzania and Uganda. This is the one in which Lakes Tanganyika and Nyasa are found. The eastern branch runs through the centre of both Kenya and Tanzania dividing each country into two halves forming important internal drainage ecosystem basins [42]. The eastern branch finally rejoins the western branch in Mbeya region fading progressively towards Lake Nyasa (Figure 1).

### Case, Outbreak and Outbreak Wave Definition

#### RVF case definition in domestic ruminants

A RVF case was suspected in domestic ruminants whenever there was a report within herd/village/district of high mortality in neonatal animals and abortion and/or stillbirths in pregnant animals. Other clinical features that were considered included reports of mucopurulent nasal discharge, bloody diarrhoea and vomiting in domestic ruminants. A probable case was considered to be a suspected case if it occurred during periods with above normal rainfall and involved widespread abortion and high mortality in neonatal domestic ruminants. In addition, the presence of haemorrhages in the internal organs of affected animals would have been reported on post-mortem.

A confirmed case was any case with laboratory confirmation of RVF by detection of specific immunoglobulin G (IgG) and/or immunoglobulin M (IgM) antibodies by ELISA. Reports on other specific tests for confirmation of RVF cases in domestic ruminants in the country were not available. In this study, both probable and confirmed cases of RVF in domestic ruminants were used collectively in the data analysis.

#### RVF case definition in humans

A confirmed case was any suspected or probable case with laboratory confirmation of RVF by detection of specific IgM antibodies by ELISA or detection of viral RNA by real-time reverse transcriptase PCR or detection of viral antigens in biopsy tissues by immunohistochemistry. No probable or suspected human cases of RVF, which fulfil clinical criteria without laboratory confirmation being done, have been identified in our data.

#### Outbreak definition

In this study, RVF outbreak was defined as occurrence in a specific location of multiple related cases likely to be caused by RVFV (both confirmed and probable cases characterized in the case definition) affecting domestic ruminants and/or humans.

#### Outbreak wave definition

A RVF outbreak wave referred to sequential reports of the outbreaks at various locations within Tanzania from date of onset of the first outbreak during a particular time period of the year until outbreaks were no longer reported in the country.

### Sources of Data

For the years 2000 to 2012, information on RVF outbreaks was gathered from the databases at MoLFD, Zonal Veterinary Investigation Centres (VICs), Ministry of Health and Social Welfare (MoHSW) and National Institute for Medical Research (NIMR) in Tanzania. Comparable records for the years 1930 to 2012 were collated from the files archived at MoLFD, zonal VICs, MoHSW and NIMR that included case report forms, laboratory registers/investigation forms, field surveillance forms, monthly and annual reports, and Food and Agriculture Organization reports. The small number of available published articles and reports describing the epidemiology and trend of RVF outbreaks in Tanzania were also used as a data source [35]. In addition, expert opinions on the historical occurrence of RVF outbreaks were obtained from NIMR, MoHSW and MoLFD. Historical weather data (monthly rainfall and mean minimum and maximum temperatures) for the years 1977 to 2008 were obtained from the Tanzania Meteorological Agency (TMA). The weather data were aggregated by seasons adapted from the TMA; 1 = October-December, 2 = January-February, 3 = March-May and 4 = June-September. The district and village-specific geo-coordinates were obtained from the local digital maps developed by the Institute of Resource Assessment, of the University of Dar es Salaam, Tanzania. Data on soil types and texture were obtained from the Mlingano Agricultural Research Institute in Tanga, Tanzania and were classified by their physical and chemical properties using the Food and Agriculture Organization (FAO) scheme [43].

### Data Preparation and Analysis

The month, year and geographical location of the districts and villages that reported RVF outbreaks between January 1930 and June 2007 were entered into a Microsoft Excel spreadsheet. Since data were obtained from various sources, location names were standardized before data were sorted and checked for consistency and duplication. The district and village-specific geo-coordinates were used to identify the locations that had similar names. Only one entry was retained for those villages with multiple entries for a particular month.

The data analysis focused on a spatial and temporal description of outbreaks at district/village and year/month resolution, respectively. Shapiro-Wilk and Shapiro-Francia tests were used to assess normality of continuous variables. Summary statistics were reported by using STATA version 12 (Statacorp, College Station, TX, USA). The study period was divided into two periods to identify potential changes in the reporting of the disease over time and space. Period one referred to time period prior to 1980 when RVF was not yet added into the list of notifiable diseases under the Ministry responsible for livestock in Tanzania. Period two referred to the time period from1980 onwards after RVF had been added into the list of notifiable diseases. The dataset was stratified annually in order to carry out separate analysis of each year and explore the temporal patterns of outbreak waves. Disease frequency was expressed as counts of disease cases per outbreak wave and cumulative number of cases between period one and period two. Outbreak curves were plotted in relation to rainfall and temperature to identify the monthly temporal pattern of cases throughout the study period.

#### Spatio-temporal cluster analysis of RVF cases

In order to identify high risk districts and villages, the location of most likely spatio-temporal clusters was investigated using the space-time permutation model [44]. In the current study we used presence-only data as no investigational surveys that could provide absence data had been conducted. The space-time permutation model only requires case data (presence-only data) for which the spatial location and time is known, but no information is needed about controls or a background population at risk [44]. The model parameters for maximum spatial and temporal window sizes were set such that a cluster could include a maximum of 50% of all cases. The number of Monte Carlo replications was set to 9999, so that the minimum detectable p-value would be 0.0001. This analysis was implemented using the SatScan 9.1 software (Information Management Services, Inc, Boston, MA, USA).

The distribution of areas that reported cases and buffering of the clusters (in kilometres) were mapped using ArcGIS 10 (ESRI East Africa). For the purpose of this study the districts that had reported RVF cases were assigned specific identification numbers before projecting them onto the map as follows: 1, Babati; 2, Dodoma; 3, Bariadi; 4, Bunda; 5, Hai; 6, Hanang; 7, Handeni; 8, Igunga; 9, Iringa-Urban; 10, Iringa-Rural; 11, Kahama; 12, Karatu; 13, Kibaha; 14, Kilindi; 15, Kilombero; 16, Kilosa; 17, Kiteto; 18, Kondoa; 19, Kongwa; 20, Korogwe; 21, Kwimba; 22, Lushoto; 23, Magu; 24, Manyoni; 25, Maswa; 26, Mbulu; 27, Meatu; 28, Monduli; 29, Moshi Rural, 30, Mpwapwa; 31, Mufindi; 32, Muheza; 3, Ngorongoro; 34, Njombe; 35, Nzega; 36, Pangani; 37, Rombo; 38, Same; 39, Serengeti; 40, Simanjiro; 41, Singida Rural; 42, Iramba; 43, Tanga Rural; 44, Ulanga; 45, Urambo; Morogoro Urban; 46 and Mvomero; 47.

#### Multivariable analysis

Logistic regression was used to analyse the association between various risk factors and the risk of outbreak occurrence at the district level. From descriptive data analysis of this study it was found that there was major temporal heterogeneity with respect to reporting of RVF in Tanzania. It was concluded that the latest outbreak wave in 2006/07 was likely to have the lowest reporting bias, and it was therefore used in the multivariable analysis. The outcome variable of interest was whether at least one outbreak involving domestic ruminants had been reported within each district during the 2006/07 outbreak wave. The risk factors included in this analysis have been suggested to be associated with occurrence of RVF [28], [45]–[50].

Data for 106 districts (88.33% of all districts) were used in this analysis. Fourteen (all urban) districts were excluded as they did not have data for all risk factors. To take account of possible nonlinear effects of continuous-scale risk factors on the logit form of the outcome variable, these variables were grouped into three contiguous categories, each representing a third of the observations. The analysis was conducted in two steps. First, all potential risk factors were screened for statistical significance at a p-value of ≤0.20 in a univariable logistic regression analysis. In the second step, these statistically significant variables were included in a multivariable logistic regression analysis based on a forward variable selection approach utilising the likelihood ratio statistic and a p-value ≤0.05. Variables not statistically significant in the univariable analysis, but with known association with RVFV activity were also included in the multivariable analysis. The variables included in the model were limited to those that did not show significant collinearity using a diagnostic cut-off value for tolerance >0.1 and variance inflation factor <10. The Mantel-Haenszel method was used to identify the effect of confounding factors. A factor was considered to have potential confounding effect if its magnitude of change was ≥25% in the coefficient estimates of other predictors. The Hosmer and Lemeshow test was used to assess the goodness of fit of the final model [51]. The discrimination ability of the final model was assessed using receiver operating characteristic curves (ROC), based on area under the curve (AUC) [52].

## Results

### Exploratory Data Analysis

From 1930 when RVF-like disease was reported for the first time in Tanzania, further outbreaks were reported in 1947, 1957, 1960, 1963, 1968, 1977/1978, 1989, 1997/1998 and 2006/2007. During this time interval a total of 10 outbreak waves (one outbreak wave in a year) of varying size and location were reported with average inter-epidemic period (IEP) of 7.9 years (range = 3–17 years). During these past outbreaks a total of 194,750 domestic ruminant cases (cattle = 54.01%; goats = 22.78% and sheep = 23.21%) were reported. A total of 309 human cases were reported during the latest outbreak in 2006/2007 and there was no documentation on cases in humans prior to 2006.

Of the 194,750 domestic ruminant cases, 140,377 (72.08%) had information about month and district of occurrence and, 55,415 (28.45%, n = 194,750) had information about month and village of occurrence been recorded. A total of 5,197 domestic ruminant cases had information on only the year and district of occurrence. A total of 63 domestic ruminant cases had information on only the year and region of occurrence. A total of 49,113 (25.22%, n = 194,750) domestic ruminant cases had information on the year without location of occurrence been recorded. All 309 human cases reported had information about month and district and 292 (94.50%) of these had information about month and village of occurrence. Of the 198 villages that were included in this study we were unable to identify the geographical coordinates for two and 16 of those that reported cases in domestic ruminants and humans, respectively. In these instances, the results were summarized at district and village and, year and month resolutions.

Out of 145,637 domestic ruminant cases that had the information on the location and time of occurrence been recorded, 14,990 (10.29%) and 130,637 (89.71%) were reported in period one and two respectively with significantly higher proportion of cases (73.11%) been reported in the eastern Rift Valley ecosystem (p = 0.03). About half (53.93%) of domestic ruminant cases were confirmed in the laboratory and 46.07% were the probable cases. All 309 humans cases were confirmed in the laboratory and nearly all (99.35%) were reported in the eastern Rift Valley ecosystem. Out of 120 districts of the Tanzania mainland, 47 (39.17%) had reported at least an outbreak involving livestock and/or humans during the study period.

**Figure 2:**
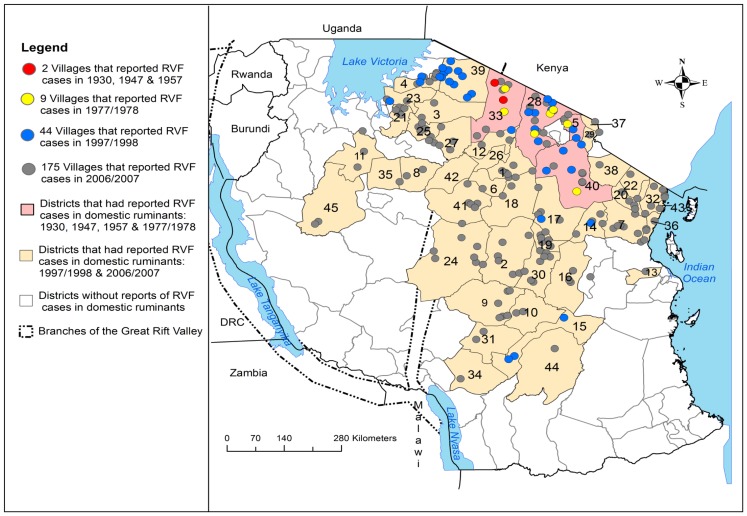
The space-time progression of Rift Valley fever outbreaks by district and villages; 1930 to 2007. Ngorongoro, Simanjiro, Monduli and Hai are the only districts in the eastern Rift Valley ecosystem that were involved in the outbreaks from 1930 to 1978. These four districts were persistently involved in subsequent outbreaks from 1997 to 2007 that had expanded progressively to east-south and western parts of the country.

The spatial distribution of the RVF cases in Tanzania varied throughout the study period. During period one (from 1930 to 1979), the cases were persistently reported in four districts in northern Tanzania. In contrast, during period two (from 1980 to 2007), spatial progression of the spread of cases from north to east-central and southern parts of the country was observed (Figure 1). Over the subsequent years, there was an increase in the number of villages that reported cases from 2 villages in 1930 to 175 villages in 2006/2007 (Table 1; Figure 2). Once the disease had been introduced into the district/village, it was likely to be involved in future outbreaks.

**Table 1:** Number of RVF cases (%) per outbreak wave by number of villages and animal species.

	Number of RVF cases (%) per outbreak wave [No. villages affected]					
Species	1930 [2]	1947 [2]	1957 [2]	1977/1978 [9]	1997/1998 [44]	2006/2007 [175]
**Cattle**	546 (53.22)	699 (49.86)	1,174 (33.37)	4,796 (42.00)	21,619 (52.94)	76,346 (55.90)
**Goats**	200 (19.49)	378 (26.96)	1,007 (28.62)	2,459 (21.57)	8,616 (21.10)	31,704 (23.21)
**Sheep**	280 (27.29)	325 (23.18)	1,337 (38.00)	4,144 (36.35)	10,600 (25.96)	28,520 (20.88)
**Total per outbreak** **wave (100%)**	**1,026**	**1,402**	**3,518**	**11,399**	**40,835**	**136,570**

From 1930 to 2007 there was a remarkable trend of increased number of ruminant cases that were reported with stability in the proportion of each animal species affected through the study period (Table 1). From 1930 to 1978 there was a 11-folds increase in the number of cases and from 1978 to 2007 there was a 12-folds further increase in the number of cases reported (Table 1). Generally during the past outbreaks, RVF cases were reported between December and June. For each of the outbreak waves from 1930 to 2007, the space-time permutation model identified statistically significant most likely and secondary clusters of cases.

According to Shapiro-Wilk and Shapiro-Francia tests, the continuous variables included into this study were not normally distributed, and therefore the median and ranges (instead of mean and standard deviation) of these variables are reported. Overall between 1930 and 2007 the number of cases per outbreak wave ranged from 1,026–136,570 with the median number of cases of 7,458.5 (25^th^ percentile = 1.402; 75^th^ percentile = 40, 835; CI = 1063.6, 126996.5). During the study period the monthly number of cases ranged from 108 to 44,367 with the median number of monthly cases of 2,281 (25^th^ percentile = 841; 75^th^ percentile = 9,814; CI = 1067.4, 6141.7). The elevation of the districts that reported RVF cases ranged from 57–1,864 m above sea level with the median elevation of 1,190 m (25^th^ percentile = 943; 75^th^ percentile = 1,445; CI = 1122, 1115).

### The Pattern of Rainfall and RVF Outbreaks

**Figure 3:**
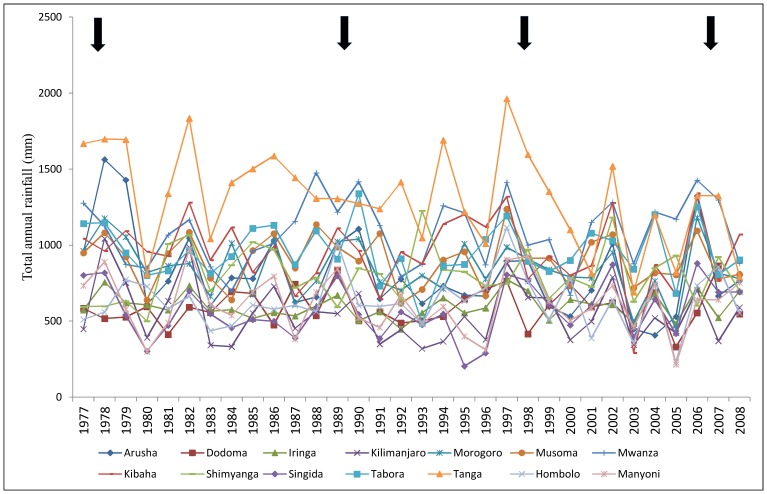
Rainfall curves showing annual total rainfall in millimetres for the zones in Tanzania. The zones represented by regions (in parenthses) are: The Northeastern zone (Arusha, Kilimanjaro, Tanga, Morogoro and Kibaha stations); Central zone (Dodoma, Hombolo, Singida and Manyoni stations); Western zone (Tabora station); Lake zone (Mwanza, Shinyanga and Musoma stations); Southern highland zone (Iringa station). The arrows indicate the years of RVF outbreak waves in Tanzania from 1977 to 2008.

The total annual rainfall ranged from 369.1–1,562.9 mm with the median total annual rainfall of 777.8 mm (25^th^ percentile = 665.8; 75^th^ percentile = 914.6; CI = 691.4, 826.1). The rainfall was not significantly different between the eastern and western Rift valley ecosystems (p = 0.52). As shown in figure 3 (arrows) the onset of past outbreaks occurred following an increased total annual rainfall compared to the previous year. The onset of the latest two outbreak waves in 2006–07 and 1997–98 occurred when the average total annual rainfall had increased by 69.97% and 51.83% respectively compared to the previous year. The onset of the 1989 and 1977–78 outbreak waves occurred when the average total annual rainfall had increased by 15.21% and 13.91% respectively compared to the previous year (Figure 3). There were other periods when the average total annual rainfall had increased by at least 22.24% compared to the previous year however, outbreaks were not reported. These year periods (together with their percentage increase in the average total annual rainfall) were; the 2003/2004 (50.77%), 2001/2002 (30.38%), 1981/1982 (28.07%), 1980/1981 (22.45%) and 1993/1994 (22.24%).

**Figure 4:**
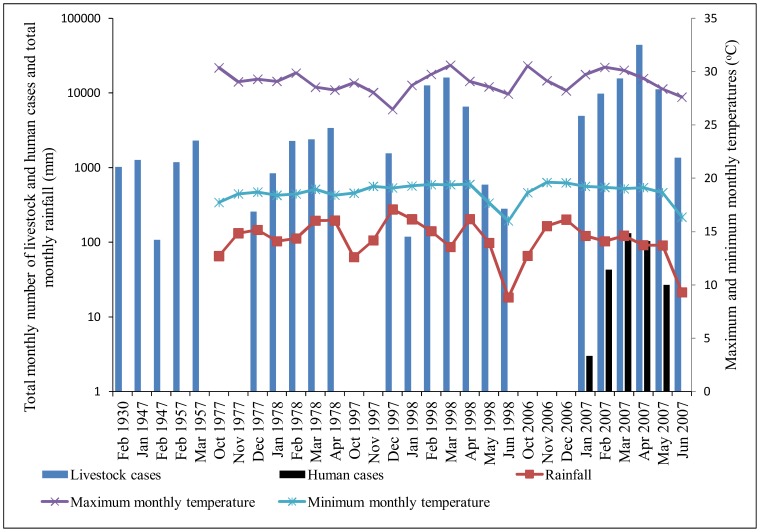
Curves showing the pattern of total monthly rainfall, temperature and RVF outbreaks waves. The onset of outbreaks in domestic ruminants [1977 to 2006/2007] and humans [2006/2007] were preceded by about two months rainful surplus. Clear pattern between the monthly tempearure and onset of outbreaks was not observed. The outbreaks had a tendency to cease as the rainfall faded-off. Left Y axis corresponds to total monthly rainfall in millimetres (mm) and total monthly number of livestock and humans cases. The right Y axis corresponds to the maximum and minimum monthly temperatures. The X axis corresponds to the months and years of RVF outbreak waves.

As shown in figure 4 the onset of the 1977/1978 RVF outbreak wave in December 1977 coincided with the total maximum monthly rainfall of 147.17 mm in season 1. The peak of this outbreak wave coincided with the maximum total monthly rainfall of 195.98 mm in April 1978 (season 2). The onset of the 1997/1998 outbreak wave in December 1997 coincided with the total maximum monthly rainfall of 275.55 mm in season 1. Although the peak of this outbreak wave in March 1998 (season 3) did not coincide with maximum total monthly rainfall, there was a tendency for the outbreak to cease as the rainfall faded-out in June 1998 (season 4). The onset of the 2006/2007 outbreak wave (in livestock and humans) in January 2007 (season 2) was preceded by the maximum total monthly rainfall of 201.45 mm in December 2006 (season 1). The peak of the livestock outbreak wave in April 2007 was preceded by the maximum total monthly rainfall of 122.33 mm in March 2007 (season 3) that coincided with the peak of human outbreak wave (Figure 4). The odds of clustering of cases was higher in season 2 (OR = 2.64, CI: 0.35, 19.88) than season 3 (OR = 1.97, CI: 0.27, 14.19) and season 4 (OR = 1.4, CI: 0.14, 13.57) although not at the 0.05 level of significance (p = 0.56).

### The Pattern of Average Annual/Monthly Maximum/Minimum Temperature and RVF Outbreaks

There was no clearly defined pattern of the average annual maximum and minimum temperatures and the RVF outbreaks (data not shown). As shown in figure 4, the optimal values of maximum and minimum monthly temperatures preceded the onset of outbreaks by at least one month. There was no clear pattern on the timing for optimal values of temperatures and the peaks of outbreaks. However, the outbreaks showed the tendency to cease as the maximum and minimum monthly temperatures declined (Figure 4).

### Spatial and Temporal Clustering of RVF Cases

**Figure 5:**
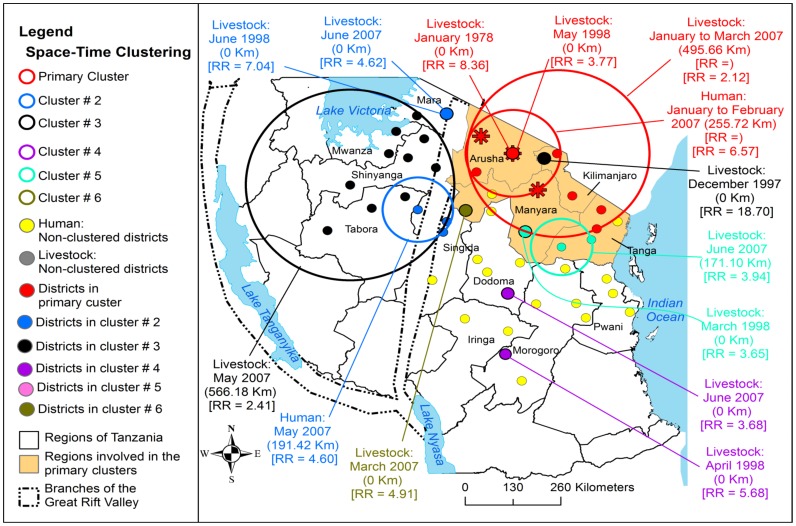
Distribution of district level space-time clusters of RVF cases in domestic ruminants and humans. The outbreak waves in 1930, 1947 and 1957 were persistently reported in one district in Arusha region. This figure indicates the persistence spatiotemporal overlapping of the livestock and human primary clusters. During the latest outbreak in 2006/2007, the primary and secondary human clusters had occurred within the respective primary and secondary livestock clusters. Asterisks correspond to districts that were included within the human primary cluster; relative risk for each cluster is displayed (RR) along with the buffer (circle) size in kilometres (km).

**Figure 6:**
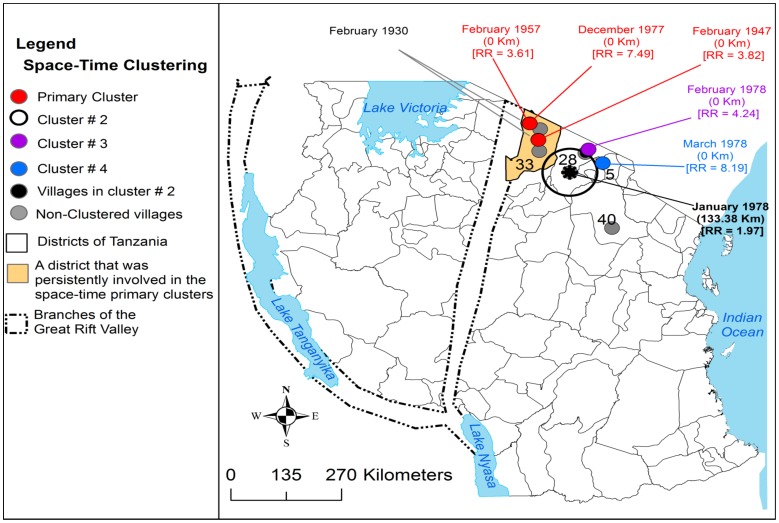
Distribution of village-level space-time clusters of RVF cases from 1947 to 1978. There were no clusters detected in 1930; from 1947 to 1978 three primary clusters were persistently detected in Ngorongoro district, each involving one village. An asterisk represents the centre of cluster that involved more than one village; relative risk for each cluster is displayed (RR) along with the buffer (circle) size in kilometres (km).

**Figure 7:**
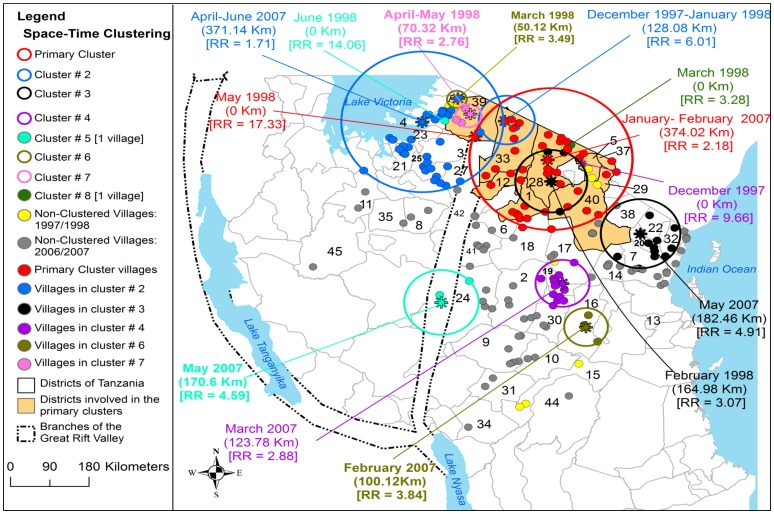
Distribution of village-level space-time clusters of RVF cases in domestic ruminants from 1997 to 2007. Asterisks represent the centre of clusters; relative risk for each cluster is displayed (RR) along with the buffer (circle) size in kilometres (km). The primary cluster was detected within the epicentre district (Ngorongoro) in the northern zone during the 1997/1998 outbreak wave. Nine districts formed the primary cluster including the epicentre district during the 2006/2007 outbreak wave that had expanded towards the south-west.

**Figure 8:**
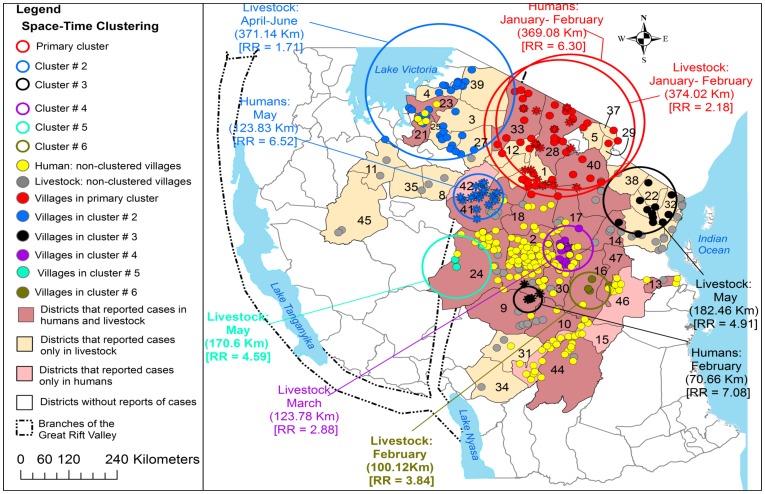
Distribution of village-level space-time clusters of RVF cases in humans and domestic ruminants. During the 2006/2007 outbreak wave the analysis of clustering of cases was made separately for humans and domestic ruminants. Between January and February 2007 there was an overlap of livestock and human primary clusters in the same location. Asterisks correspond to villages that were included within human space-time clusters; Relative risk for each cluster is displayed (RR) along with the buffer (circle) size in kilometres (km).

From 1930 to 2007 the clustering of RVF cases was persistently and predominantly detected in the eastern Rift Valley ecosystem of the country compared to the western ecosystem (Figure 5, 6, 7 and 8). The odds of clustering of RVF cases was higher in the eastern Rift Valley ecosystem than the western ecosystem (OR = 1.76, CI: 0.89, 3.47), although not at the 0.05 level of significance (p = 0.10). Out of 46 districts that had reported outbreaks in domestic ruminants in the past, 32 (69.57%) had reported at least one cluster of cases and 13 (28.26%) had no clustering of cases. Ngorongoro district in northern Tanzania was persistently involved in the clustering of cases throughout the study period. The spatio-temporal overlapping of the primary and secondary livestock and human clusters was observed during the study period (Figure 5, 6, 7 and 8). The space-time clustering of livestock and human cases showed a tendency to spread from the north to east-central and western parts of the country (Figure 5, 6, 7 and 8).

### District-level Clustering of Cases

The outbreak waves of 1930, 1947 and 1957 were reported in Ngorongoro district. During the 1977–788 and 1997–98 outbreak waves the district-level primary clusters were detected in Monduli in Arusha region with respective relative risk of 8.36 (in January 1978) and 3.77 (in May 1998) (Figure 5). During the 1997–98 outbreak wave a total of four secondary clusters were detected in Arusha, Kilimanjaro, Mara, and Morogoro regions (Figure 5). During the 2006–07 outbreak wave a larger livestock primary cluster (diameter of 495.66 km) was detected between January and March 2007. A total of 10 districts (including the district that was the primary cluster during the 1977–78 and 1997–98 outbreak waves) formed the primary cluster of the 2006/2007 outbreak wave (Figure 5). A relatively smaller human primary cluster (diameter of 255.72 km) was detected within the livestock primary cluster between January and February 2007 with a relative risk of 6.57 (Figure 5). During this outbreak wave one secondary human cluster with a diameter of 191.42 km and a relative risk of 4.60 was detected within the relatively bigger secondary cluster of livestock (diameter = 566.18 km; relative risk = 2.41) and both were detected in May 2007 (Figure 5).

### Village-level Clustering of Cases

Three village-level primary clusters were detected in one district in northern Tanzania during the 1930, 1947 and 1957 outbreak waves (Figure 6). Two primary clusters were detected in the same region during the 1997–98 (relative risk of 17.33) and 2006/2007 (relative risk of 2.18) outbreak waves (Figure 7). The primary cluster that was detected during 1997/1998 outbreak wave became part of the relatively larger primary cluster that was detected during 2006–07 outbreak wave (Figure 7). This figure indicate further that, two of the secondary clusters that were detected during 1997–98 outbreak wave were included in the primary cluster detected during 2006–07 outbreak wave.

### Descriptive Analysis of Specific RVF Outbreak Waves

#### RVFV activity during the 1930 outbreak wave

During this outbreak wave a total of 1,026 (0.53%, n = 194,750) domestic ruminant cases were reported in two villages in 1/120 (0.83%) districts of Tanzania Mainland. Ngorongoro was the only district that reported cases during this outbreak wave (Figure 2) in February (Figure 4). Clustering of cases was not detected during this period (Figure 5 and 6). Relatively larger proportion of cattle (53.22%) was affected followed by sheep (27.29%) and goats (19.49%) (Table 1).

#### RVFV activity during the 1947 outbreak wave

During this outbreak wave a total of 1,402 (0.72%, n = 194, 750) domestic ruminant cases were reported in two villages (the same that had reported cases during the 1930 outbreak wave) in Ngorongoro district (Figure 2) from January to February with peak in January (Figure 4). The monthly median number of cases was 688.5 (range: 108–1269; 25^th^ percentile = 108; 75^th^ percentile = 1,269). A primary cluster was detected in one village in Ngorongoro district in February with a relative risk of 3.82 (Figure 6). A relatively larger proportions of cattle (49.86%) was affected followed by goats (29.96%) and sheep (23.18%) [Table 1].

#### RVFV activity during the 1957 outbreak wave

During this outbreak wave a total of 3,518 (1.81%, n = 194,750) domestic ruminant cases were reported in two villages (the same that had reported cases during the 1947 outbreak wave) in Ngorongoro district (Figure 2) from February to March with peak in March (Figure 4). The monthly median number of cases was 1,740 (range: 1184–2,296; 25^th^ percentile = 1,184; 75^th^ percentile = 2,296). A primary cluster was detected in one village in Ngorongoro district in February with a relative risk of 3.61 (Figure 5). Relatively larger proportion of sheep (38.00%) was affected followed by cattle (33.37%) and goats (28.62%) (Table 1).

#### RVFV activity during the 1960, 1963, 1968 and 1989 outbreak waves

Limited information available in a consultancy report from a field mission of the Food and Agriculture Organization of the United Nations indicated that, RVFV had been isolated in Tanzania in 1960, 1963, 1968 and 1989. This report did not provide details on the specific outbreak wave, number of cases per month, year and districts or villages affected. It however, indicates that these outbreaks mainly affected exotic breeds of cattle and sheep where it resulted in abortion storms, mortality in young animals and drop in milk production associated with fever. The report indicate further that, RVFV was isolated from these animals in Ngorongoro, Mpwapwa, Morogoro Rural, Mufindi, Moshi Rural, Hai and Iringa Rural districts without details of number of isolates. These outbreaks are reported in our study but were not included in the analysis.

#### RVFV activity during the 1977/1978 outbreak wave

Exploration of available data for 1977/1978 indicated that the outbreak had spread to include nine villages (number of villages in parentheses) in 4/120 (3.33%) districts; Ngorongoro (4), Monduli (3), Simanjiro (1) districts and Hai district (1) (Figure 2). In 1977 cases were reported in December and in 1978 cases were reported from January to March with the peak in January (Figure 4). During this outbreak wave a total of 11,399 (5.85%, n = 194,750) domestic ruminant cases were reported (Table 1). The monthly median number of cases was 2,281 (range: 257–3,413; 25^th^ percentile = 841; 75^th^ percentile = 2,389). The two villages that had previously reported cases were also involved in this outbreak. The district-level most likely cluster was detected in Monduli in January 1978 with a relative risk of 8.36 (Figure 5). The village-level most likely cluster was detected in one village in Ngorongoro district in December 1977 with a relative risk of 7.49 (Figure 6). Three statistically significant village-level secondary clusters were detected during this outbreak wave (Figure 6). Relatively larger proportion of cattle (42.00%) was affected followed by sheep (36.35%) and goats (21.57%) (Table 1).

#### RVFV activity during the 1997/1998 outbreak wave

A relatively large outbreak wave in 1997/1998 involved 44 villages (number of villages in parentheses) in 9/120 (7.50%) districts; Serengeti (11), Monduli (11), Ngorongoro (6), Simanjiro (4) and Kiteto (2), Bunda (3), Hai (3), Kilombero (3) and Magu (1) (Figure 2). In 1997, RVF cases were reported in December and in 1998 cases were reported from January to June with the outbreak reaching its peak in March 1998 (Figure 4). During this outbreak wave a total of 40,835 (29.90%, n = 194,750) domestic ruminant cases were reported (Table 1). The monthly median number of cases during the wave of this outbreak was 1,554 (range: 119–16,175; 25^th^ percentile = 281; 75^th^ percentile = 12,612). The four districts and nine villages that had previously reported cases during the 1977/1978 outbreak wave were also involved in this outbreak. The most likely district-level cluster of domestic ruminant cases was detected in Monduli in May 1998 with a relative risk of 3.77. A total of four statistically significant district-level secondary clusters were detected in Serengeti, Hai, Kilombero and Kiteto (Figure 5). At village level, the most likely cluster of domestic ruminant cases was detected in one village in Serengeti district in May 1998 with a relative risk of 17.33 (Figure 7). A total of seven statistically significant village-level secondary clusters were detected during this outbreak wave (Figure 7). Relatively larger proportion of cattle (52.94%) was affected followed by sheep (25.96%) and goats (21.10%) (Table 1).

#### RVFV activity during the 2006/2007 outbreak wave

During this outbreak wave, documentation on RVF cases in domestic ruminants and humans was available and some villages had concurrently reported RVF in livestock and human populations.

The widely spread outbreak wave in 2006/2007 in domestic ruminants involved 175 villages in 45/120 (37.50%) districts in north-eastern and central Tanzania (Figure 2). Seven districts and twenty one villages that had reported cases during the previous outbreak waves were also involved in this outbreak (Figure 2). RVF cases were reported from January to June with a peak in April (Figure 4). A total of 136, 570 (70.13%, n = 194, 750) domestic ruminant cases were reported during this outbreak wave (Table 1). The monthly median number of domestic ruminant cases during the wave of this outbreak was 10,513 (range: 1,356–44,367,175; 25^th^ percentile = 4,932; 75^th^ percentile = 15,726). Relatively larger proportion of cattle (55.90%) was affected followed by goats (23.21%) and sheep (20.88%) (Table 1).

The district-level most likely cluster of domestic ruminant cases was detected between January and March 2007 with 21, 092 cases. This cluster had involved 10 districts namely Simanjiro, Monduli, Ngorongoro, Karatu, Kiteto, Rombo, Same, Korogwe, Lushoto, and Babati with a diameter of 495.66 km and relative risk of 2.12. A total of 5 statistically significant district-level secondary clusters were detected during this outbreak wave (Figure 5). The village-level most likely cluster of domestic ruminant cases involved 44 villages and was detected between January and February 2007 with 9,704 cases, a diameter of 374 km and relative risk of 2.18 (Figure 7). The primary cluster that was detected during the 1997/1998 outbreak wave became part of the 2006/2007 primary cluster (Figure 7). A total of five statistically significant village-level secondary clusters were detected (Figure 7). Two secondary clusters that were detected in December to January in 1997/1998 and February 1998 were embedded within the 2006/2007 primary cluster (Figure 7).

During 2006/2007 outbreak wave, a total of 309 human cases were reported in 210 villages in 28/120 (23.33%) districts of which 21 (75.00%) districts concurrently had reported cases in humans and domestic ruminants. A total of 163 (52.75%) human cases were reported in Dodoma region with relatively larger number of cases been reported in Dodoma Rural (43.56%) and Dodoma Urban (36.81%) districts. Generally human RVF cases were spatially concentrated in the central zone of the country (Figure 8). The monthly median number of human cases during the wave of this outbreak was 43 (range: 3–132; 25^th^ percentile = 27; 75^th^ percentile = 104). The human cases were reported from January to May with the peak in March and no cases were reported beyond May (Figure 4). During this outbreak wave the most likely cluster of human cases included seven districts and 16 villages. This cluster was detected between January and February 2007 with 17 cases, diameter of 369.8 km and relative risk of 6.3.

It was interestingly to observe that, the primary clusters of human and domestic ruminant cases were detected in the same location and time during the 2006/2007 outbreak wave (Figure 5 and 8). One district-level and two village-level statistically significant secondary clusters were detected (Figure 5 and 8). The secondary human and livestock space-time clusters showed a tendency to spread from north to western and east-central parts of the country (Figure 5 and 8). The onset of outbreaks in both the livestock and humans occurred in January with human to livestock case ratio of 1 to 1644. During this outbreak wave the overall ratio of human to livestock cases was 1 to 442.

### Risk Factor Analysis

#### Univariable logistic regression analysis

The univariable data analysis showed that the districts that experienced a bimodal rainfall pattern were more likely to report RVF cases than other districts (Table 2). This analysis suggested further that reports of cases within the districts were positively associated with cattle density of >13.5 heads per km^2^; sheep density of >6.17 heads per km^2^; goat density of >6.3 heads per km^2^; pastoral livelihood zone; close to open grassland cover, clay and loam soil texture (Table 2). Outbreak occurrence was more likely to be preceded by a total monthly rainfall of >176.4 mm during each of the previous two months and total monthly rainfall of >133.8 mm during the previous month. Total amounts of rainfall during the previous 2 months >405.4 mm and during the previous 3 months of >313.4 mm were associated with outbreak occurrence in the district.

**Table 2:** Results from univariable logistic regression analysis of potential risk factors for Rift Valley fever outbreak occurrence in domestic ruminants at district level in Tanzania, during the 2006/2007 outbreak wave.

Variable	OR	95% CI	P-value	Variable	OR	95% CI	P-Value
Livestock density(number per km^2^)				Cumulative rainfall (mm)			
Cattle				Sum of last two months			
≤13.5	Ref.			≤257.7	Ref.		
>13.5–39.82	4.37	1.52, 12.64	0.006	>257.7–405.4	2.38	0.86, 6.64	0.096
>39.82	5.52	1.91, 15.99	0.002	>405.4	7.00	2.41, 20.37	0.002
Sheep				Sum of last three months			
≤1.22	Ref.			≤313.4	Ref.		
>1.22–6.17	2.07	0.73, 5.87	0.172	>313.4–501	3.22	1.22, 8.48	0.018
>6.17	7.64	2.64, 22.07	0.002	>501.1	6.13	1.90, 19.76	0.002
Goats				**Elevation (metres** **above sea level)**			
≤6.3	Ref.			≤887	Ref.		
>6.3–28.63	3.18	1.16, 8.67	0.024	>887–1187	1.27	0.49, 3.31	0.626
>28.63	3.18	1.16, 8.67	0.024	>1187	1.43	0.55, 3.70	0.467
**Rift Valley ecosystem**				**Livelihood zones**			
Western Rift Valley	Ref.			Others	Ref.		
Eastern Rift Valley	2.08	0.92, 4.73	0.078	cotton-paddy-cattle midlands	2.24	0.73, 6.86	0.157
**Rainfall pattern**				Pastoral zone	7.17	1.84, 28.06	0.004
Unimodal	Ref.			**Soil types**			
Bimodal	2.66	1.16, 6.10	0.021	Others	Ref		
**Total monthly** **rainfall (mm)**				Acrisols	2.54	0.59, 10.95	0.211
Three months ago				Cambisols	0.56	0.22, 1.37	0.200
≤12.2	Ref.			Ferralsols	0.13	0.57, 1.94	0.876
>12.2–55.7	0.21	0.07, 0.64	0.006	**Soil texture**			
>55.7	1.79	0.69, 4.65	0.231	Sandy	Ref.		
Two months ago				Clay	9.20	2.81, 30.12	0.001
≤102	Ref.			Loam	6.19	1.63, 23.42	0.007
>102–176.4	1.41	0.52, 3.84	0.498	**Land cover**			
>176.4	4.23	1.55, 11.55	0.005	Others	Ref.		
One month ago				Closed broadleaved deciduous forest	2.32	0.53, 10.04	0.262
≤133.8	Ref.			Closed to open grassland	8.25	1.45, 46.86	0.017
>133.8–210	8.06	2.72, 23.90	0.000	Mosaic Vegetation/Croplands	2.85	0.70, 11.54	0.142
>210	3.98	1.29, 12.30	0.016				

#### Multivariable logistic regression analysis

Results of the multivariable logistic regression analysis are summarized in Table 3. Three of 15 variables were included in the final multivariable logistic regression model; eastern Rift Valley ecosystem versus western Rift Valley ecosystem in Tanzania (OR = 6.14, CI: 1.96, 19.28), a total amount of rainfall during the previous two months >405.4 mm (OR = 12.36, CI: 3.06, 49.88), soil texture (clay [OR = 8.76, CI: 2.52, 30.50] and loam [OR = 8.79, CI: 2.04, 37.82]). There was no statistical evidence of collinearity between these risk factors. Confounding factors were not observed during the model building process. The Hosmer-Lemeshow statistic suggested a good fit to the data (chi2 = 7.29, 8 df, p = 0.51). The assessment of the predictive accuracy of the multivariable model based on the area under the curve (AUC) derived from the receiver operating characteristic curve analysis (AUC = 0.84) suggested that the final model provided good discrimination.

**Table 3:** Final multivariable logistic regression model for Rift Valley fever occurrence at district level in Tanzania during the 2006/2007 outbreak wave.

Rift Valley ecosystem	Odd Ratio	95% CI	P-value
Western Rift Valley	Ref.		
Eastern Rift Valley	6.14	1.96, 19.28	0.002
**Cumulative rainfall (mm)**			
Sum of last two months			
≤257.7	Ref.		
>257.7–405.4	2.38	0.76, 7.48	0.137
>405.4	12.36	3.06, 49.88	0.001
**Soil texture**			
Sandy	Ref.		
Clay	8.76	2.52, 30.50	0.001
Loam	8.79	2.04, 37.82	0.004

## Discussion

The spatial and temporal pattern of RVF outbreaks has not previously been described in Tanzania. It is interesting to note that the circumstances that allow the onset of sequential RVF outbreaks often prevail simultaneously throughout the eastern and southern African countries [28],[45]–[50]. Although epidemiological features of RVF in Tanzania do not seem to be fundamentally different compared with the neighbouring countries, it is unique that, this is the only country with the two branches of the Great Rift Valley (that is associated with RVF occurrence). The Great Rift Valley branches in the north of the country as it traverses from Kenya forming the important eastern and western internal drainage ecosystems in Tanzania before finally rejoining in the southern highland of the country.

Results of our analysis indicate that most of the districts and villages that were involved in past outbreaks were located in northern Tanzania, the eastern wing of the Great Rift Valley, and that transmission was seasonal. In this study, a general trend of RVF outbreaks spreading from north to east-central and southern parts of the country was observed between 1930 and 2007. During the first 27 years following the first report of RVF-like disease in Tanzania, the disease was only reported from two villages in the Ngorongoro district where livestock are raised in proximity to wildlife. Subsequent outbreak waves included an increasing number of villages, districts and regions in Tanzania which had never reported outbreaks. The low numbers of reported cases of RVF in the early years (1930 to 1978) before the disease was added in the list of notifiable diseases) could be attributed to poor awareness about the disease, inefficient recording/reporting systems and lack of diagnostic capacity. The surveillance activities were more comprehensive during the last outbreak in 2006–07 than during the previous outbreaks. These findings should therefore be interpreted cautiously due to the fact that the disease might have been present for some years before it was first diagnosed and may not have been detected in other potentially affected areas. It seems reasonable to assume, however, that initial amplification in the northern zone of Tanzania generated a source of risk which resulted in progressive infiltration of the disease to the rest of the country. Although the mechanisms of the spatial spread are not known, they are likely to include active and passive movement of infected mosquitoes and uncontrolled livestock movements within the country. However, data on the distribution of potential vectors and livestock movement pattern is Tanzania is scarce.

The spatiotemporal progression of RVF outbreaks described in this study had a similar trend to that in neighbouring country Kenya [49]. In Tanzania, between 1930 and 1957 only less than 1% of the districts were constantly involved in the outbreaks. The 1977–78 outbreak wave had involved 4/120 (3.33%) districts. A relatively larger outbreak wave in 1997–98 involved 7.70% of the districts and the widely spread outbreak wave in 2006–07 involved humans and domestic ruminants in 39.17% of the districts in the country. The outbreak wave of 1951 in Kenya involved 8/69 (12%) administrative districts. Between 1961 and 1964 the outbreaks had expanded to include 32% districts across six provinces including all of the districts that had reported cases previously. The latest outbreak in 2006/2007 was most extensive and had involved 48% of the districts in Kenya [49].

Our findings indicate that the outbreak waves of 1977/1978, 1989, 1997/1998 and 2006/2007 were preceded with periods of positive surplus rainfall. Furthermore, the findings suggest that the outbreak waves were likely to end as the rainfall and warm temperatures faded-out. Compared to the eastern Rift Valley ecosystem; the western Rift Valley ecosystem received the unimodal rainfall and was less likely to report outbreaks and clustering of cases. The relationship between RVF outbreaks and rainfall has been indicated before [45]–[50]. However, there was no significant difference in the amount of rainfall between the two ecosystems during the study period. Furthermore, there were instances where no outbreaks were recorded following the seasons of exceptionally above normal rainfall. These observations suggest that while rainfall might be the major determinant for the onset and switch-off of an outbreak, it is likely to be not the only factor responsible for the spread and clustering of RVF cases. A causal association between local environmental factors, livestock density and movement, encroachment of mosquitoes into new areas and occurrence of RVF has been suggested in the previous studies [53]–[56]. Anecdotal reports also suggest that levels of herd immunity might be responsible for modifying temporal patterns of RVFV occurrence.

The multivariable model demonstrates the collective effect of the Rift Valley ecosystem, cumulative effect of the amount of rainfall and soil texture on the RVF occurrence within districts. Previous studies had reported the association of some of these factors and RVF outbreaks [45]–[50]. Probably the eastern Rift Valley ecosystem provides suitable ecological features necessary for livestock keeping and survival of RVFV. This ecosystem experiences the bimodal rainfall pattern. It seems plausible to suggest that the occurrence of RVF outbreaks is associated with certain amount of rainfall. The increased rainfall preceding the onset of an outbreak provides an environment for *Aedes* and other mosquito species to emerge in large numbers with the resulting extensive transmission of the virus to animals and humans. The clay and loam soil texture support long period retention of water contributing to flooding and wetness habitat suitable for breeding and survival of the mosquito vectors. Uncontrolled livestock movement might have been responsible for the observed spatio-temporal spread of the outbreaks from north to east-central and southern parts of the country. These hypothesized mechanisms of disease occurrence and spread in the study areas need further investigation.

On the other hand, the district outbreak reporting status might be affected by the temporal dependence of the risk factors and implementation of surveillance and control activities. Heterogeneity was observed in the reporting of cases between districts over time. Although extensive surveillance and control measures were implemented during the last outbreak wave of 2006–07, it is not possible to account for variations and extent of surveillance activities between districts that might influence the disease reporting status of the district. The increased risk of RVF occurrence in the eastern Rift Valley ecosystems reported in this study is supported by the findings of the recent inter-epidemic sero-surveillance demonstrating higher prevalence of antibodies to RVFV in the eastern than the western Rift Valley ecosystem (C. Sindato et al., unpublished data). Past RVF outbreaks in Tanzania tend to cease as the rainfall faded-off, consequently it is difficult to conclude on the success of emergency vaccination programmes usually implemented too late during RVF outbreaks. Therefore, specific studies should be carried out to monitor and evaluate the effectiveness of the vaccination strategies.

Our findings further suggest that, once RVFV had been introduced to a new geographical area, it becomes endemic. These newly established endemic areas constitute a source for future outbreaks once favourable environmental conditions allow for re-activation of large scale virus transmission. A similar observation was reported in Kenya [49]. In this study we identified areas with persistence risk and clustering of RVF cases in humans and livestock. The onset of outbreaks in both the livestock and humans in 2006/07 occurred in January with human to livestock case ratio of 1 to 1644. This suggests that, for a single human case to be reported, a certain level of disease incidence in livestock may be necessary. The pattern of RVF outbreak waves in livestock and human populations and spatio-temporal overlapping of both populations’ primary clusters, suggest that there is a strong relationship between human and livestock outbreaks. A similar observation was reported in Kenya where the clustering of human RVF cases occurred around livestock cases that was preceded by the onset of outbreak in the latter [50]. In our study, concluding whether the onset of outbreak in livestock preceded the outbreak in humans or the *vice versa* is however, limited since the specific date of the onset of livestock outbreak was rarely recorded. Likewise, largely the district and village of outbreak origin were not recorded, and the specific onset of human outbreaks was difficult to determine from data passively captured by health facilities. Although the human clusters were of relatively smaller size and were likely to be detected within the relatively bigger livestock clusters, the overlapping of the primary clusters in the same location and time period make it difficult to determine the exact interval between the onset of outbreaks in livestock and humans. Improved surveillance and inter-sectoral collaboration during outbreak investigation would improve the quality of data in future.

Based on the reported data for the last disease outbreak in 2006–07, it appears that in central Tanzania humans were more likely to be affected compared with other areas of the country. This is a surprising observation for a zone in which no RVF outbreaks have ever been reported before. While the reasons are not known, it is likely that RVFV persisted in transovarially infected eggs of floodwater *Aedes* mosquito species or circulated at sub-epidemic levels between vectors and susceptible animals until anomalous high rainfalls leading to massive flooding and the resultant swarms of competent mosquito vectors triggered transmission of the virus to a wide range of susceptible vertebrate species [12]. Other factors that might have contributed to amplification of cases in central Tanzania could have been introduction of infected animals and behavioural risk practices including the human consumption of meat from carcasses that had not been inspected or were from dead animals (L.E.G. Mboera *et al*. unpubl. data). Given that the majority of human RVF cases are caused by animal-human transmission there is a need to examine the social and economic factors that may differentiate these sites from others. Furthermore, the observed spatial RVF outbreaks in humans coincide with malaria outbreaks reported previously in northern (Ngorongoro, Babati, Hanang and Mbulu Districts) and central (Dodoma, Mpwapwa and Kongwa Districts) Tanzania [57] suggesting that RVF cases might have been misdiagnosed. For instance, during the latest outbreak in 2006–07 the majority of cases in central Tanzania were initially admitted as malaria or psychiatric cases but later confirmed to be RVF cases (L. E. G. Mboera *et al*. unpubl. data).

Our findings suggest that there is continuous endemicity of RVFV in some areas making them vulnerable to periodic outbreaks, an observation that collaborate with results from previous studies in Kenya [49] and South Africa [58]. For example, Ngorongoro district was involved in the outbreak wave of 1930 and it was involved in all the subsequent outbreaks that had occurred in the country. Our results suggest that there has been the recurrence of disease in this district and that the district has remained the index foci during outbreaks before the disease is reported elsewhere in the country. RVF cases were reported only in this district between 1930 and 1957. Ngorongoro district borders Kenya in the north and humans, wild and domestic animals are able to mix freely in this district. Available data on the role of wildlife in the epidemiology of RVF is limited. Serological evidence of RVFV activity has been reported in wildlife in Zimbabwe and Kenya [59], [60]. Of recently the RVF activity has been detected in limited samples of African buffalo and elephant collected during the IEP in Tanzania [61]. The role of wildlife in the maintenance and transmission of RVFV in the country therefore requires further investigation.

The observed geographical spread of RVF over the period from 1930 until 2007 examined in this study provides evidence that the next outbreak may well spread rapidly to large populations of humans and domestic animals, potentially even involving the entire country. The reported clusters defined as high risk areas should be targeted with strategic control measures including targeted livestock vaccination and public health education especially during the IEP. The priority for strategic control programme should target the recent human and livestock overlapping primary and secondary space-time clusters. This strategy would allow more cost-effective usage of limited resources that can help to control future outbreaks/spread and the associated disease health and socio-economic impacts. The currently in use live attenuated veterinary vaccine based on the Smithburn neurotropic strain (SNS) of the virus strain should not be used once the outbreak starts to minimize the risk of needle spread of outbreak virus [62]. It is worth mentioning that recent results of molecular study by Grobbelaar et al. [13] suggest that the natural history of RVFV and its pathogenicity to humans might be influenced by massive vaccination of ruminants in Africa with the live attenuated SNS vaccine.

This study has some important limitations. First, although under-reporting and misdiagnosis of cases during the past outbreaks might have contributed to the observed pattern, we are unable to unveil a possibility of more cases to have been reported during the latest outbreak wave due to increased levels of awareness, surveillance and advocacy. For example in the 2006/07 outbreak, there was more extensive livestock surveillance than in any of the previous outbreaks. Second, even though our study has shown a non-random distribution of cases, data from some surveillance systems can manifest significant non-random geographic distribution because of variability not only in disease incidence, but also in diagnosis and reporting, factors which are strongly affected by human socioeconomic activities/behaviour and could not be measured or controlled for in our analyses. Furthermore, RVF cases in domestic ruminants were not always confirmed in the laboratory during past outbreaks and this may have resulted in over-reporting of the number of cases per district/village. However, while investigating the epidemiological trend of outbreaks in Tanzania, the focus was made to the years that corresponded to periods of past outbreaks that had occurred in the neighbouring country Kenya. We believe that the findings of this study illustrate the need to improve inter-sectoral collaboration, diagnosis, reporting and recording of disease events in the country as well as cross-border surveillance. This study offers a useful baseline description of apparent spread of RVF risk in the country. Despite its limitations, our investigation provides important findings which should be used to influence research priorities, policy development and allocation of disease control/management resources.

## Conclusion

The RVF outbreaks were found to be distributed heterogeneously and transmission dynamics appeared to vary even between areas within a few kilometres apart. The sequence of outbreak waves, continuously cover more parts of the country. Whenever infection has been introduced into an area, it is likely to be involved in future outbreaks. During the 2006/07 outbreak wave, cases were more likely to be reported from the eastern Rift Valley than the western Rift Valley ecosystem and in areas with clay and loam soil than sandy soil texture. The findings demonstrate the value of retrospective spatio-temporal analysis for informing the planning and implementation of strategic control measures.
